# Alterations in theta-gamma coupling and sharp wave-ripple, signs of prodromal hippocampal network impairment in the TgF344-AD rat model

**DOI:** 10.3389/fnagi.2023.1081058

**Published:** 2023-03-22

**Authors:** Monica van den Berg, Daniëlle Toen, Marleen Verhoye, Georgios A. Keliris

**Affiliations:** ^1^Bio-Imaging Lab, University of Antwerp, Wilrijk, Belgium; ^2^μNEURO Research Centre of Excellence, University of Antwerp, Antwerp, Belgium; ^3^Institute of Computer Science, Foundation for Research and Technology – Hellas, Heraklion, Crete, Greece

**Keywords:** Alzheimer’s disease, synaptic dysfunction, electrophysiology, hippocampus, TgF344-AD rat model, sharp wave-ripple, phase-amplitude coupling

## Abstract

Alzheimer’s disease (AD) is a severe neurodegenerative disorder caused by the accumulation of toxic proteins, amyloid-beta (Aβ) and tau, which eventually leads to dementia. Disease-modifying therapies are still lacking, due to incomplete insights into the neuropathological mechanisms of AD. Synaptic dysfunction is known to occur before cognitive symptoms become apparent and recent studies have demonstrated that imbalanced synaptic signaling drives the progression of AD, suggesting that early synaptic dysfunction could be an interesting therapeutic target. Synaptic dysfunction results in altered oscillatory activity, which can be detected with electroencephalography and electrophysiological recordings. However, the majority of these studies have been performed at advanced stages of AD, when extensive damage and cognitive symptoms are already present. The current study aimed to investigate if the hippocampal oscillatory activity is altered at pre-plaque stages of AD. The rats received stereotactic surgery to implant a laminar electrode in the CA1 layer of the right hippocampus. Electrophysiological recordings during two consecutive days in an open field were performed in 4–5-month-old TgF344-AD rats when increased concentrations of soluble Aβ species were observed in the brain, in the absence of Aβ-plaques. We observed a decreased power of high theta oscillations in TgF344-AD rats compared to wild-type littermates. Sharp wave-ripple (SWR) analysis revealed an increased SWR power and a decreased duration of SWR during quiet wake in TgF344-AD rats. The alterations in properties of SWR and the increased power of fast oscillations are suggestive of neuronal hyperexcitability, as has been demonstrated to occur during presymptomatic stages of AD. In addition, decreased strength of theta-gamma coupling, an important neuronal correlate of memory encoding, was observed in the TgF344-AD rats. Theta-gamma phase amplitude coupling has been associated with memory encoding and the execution of cognitive functions. Studies have demonstrated that mild cognitive impairment patients display decreased coupling strength, similar to what is described here. The current study demonstrates altered hippocampal network activity occurring at pre-plaque stages of AD and provides insights into prodromal network dysfunction in AD. The alterations observed could aid in the detection of AD during presymptomatic stages.

## 1. Introduction

Alzheimer’s disease (AD) is a severe neurodegenerative disorder characterized by the progressive accumulation of extracellular amyloid-beta (Aβ) plaques, intracellular tau-aggregation, synaptic dysfunction, and neuronal loss, resulting in cognitive dysfunction and eventually, dementia. Despite decades of research, disease-modifying therapies are still lacking, mainly due to incomplete insights into the neuropathological mechanisms of AD. Studies have demonstrated that the neuropathological changes underlying AD start 10–20 years before the onset of cognitive symptoms, with the accumulation of Aβ being the first identifiable hallmark (Braak and Braak, 1991; Braak et al., 2011; Sperling et al., 2011). The soluble Aβ species, which are the precursors of the Aβ plaques, are known to interfere with synaptic dysfunction and therefore network function, long before cognitive symptoms are apparent (Busche et al., 2012; Ma and Klann, 2012; Ben-Nejma et al., 2019; Hector and Brouillette, 2020; Li and Selkoe, 2020). Research has demonstrated that imbalanced synaptic function drives disease progression during presymptomatic stages of AD, making this an interesting target for future therapies (Witton et al., 2016; Styr and Slutsky, 2018; Bi et al., 2020).

The hippocampus is a brain structure in the medial temporal lobe, which is strongly involved in memory processes. Moreover, the hippocampus is one of the first regions affected in AD (Braak and Braak, 1991; Braak et al., 2011). The main function of the hippocampus is consolidation and retrieval of memories, by integrating of sensory information with previously encoded information. This process can be observed mainly in the tri-synaptic hippocampal circuit, where the activity of the pyramidal excitatory neurons in the CA1 and CA3 layers is balanced by inhibitory interneurons (Ego-Stengel and Wilson, 2010; Buzsaki, 2015). This results in various hippocampus specific oscillatory characteristics, such as sharp wave-ripple (SWR) and phase amplitude coupling (PAC).

SWR are fast oscillatory events which occur mainly during quiet wakefulness and during sleep (Buzsaki, 2006; Buzsaki, 2015). SWR are the hallmark of memory replay, as during SWR, large population of pyramidal neurons are activated, often in sequences that recapitulate past or potential future experiences (Buzsaki, 2015; Colgin, 2016). The sharp waves and ripples are thought to be separate events with distinct origins. The sharp waves are excitatory events originating from the CA3, which induce locally generated ripple oscillations in the CA1 pyramidal layer. The ripple events are generated by a delicate interaction between excitatory pyramidal neurons and local GABAergic interneurons that, if disrupted, can lead to pathological forms of activity which impair memory processes (Buzsaki et al., 2003; Buzsaki, 2015; Colgin, 2016; Caccavano et al., 2020; Sanchez-Aguilera and Quintanilla, 2021; Zhen et al., 2021). During SWR, slow gamma oscillations have been observed throughout the entire hippocampus. These slow gamma oscillations are thought to synchronize oscillatory activity across the hippocampus, which enables memory activation (Carr et al., 2012; Gillespie et al., 2016; Sanchez-Aguilera and Quintanilla, 2021). Numerous studies have demonstrated that hyperactivity in the hippocampal circuits is associated with memory deficits at later stages of AD in different mouse models of AD (Wilson et al., 2005; Yassa et al., 2010; Bakker et al., 2015; Setti et al., 2017; Donegan et al., 2019; Ratner et al., 2021).

The amplitude of the hippocampal gamma rhythm is modulated by the phase of theta, a phenomenon named phase-amplitude coupling (PAC). The strength of this coupling has been shown to be increased during learning. Moreover, the PAC strength is also directly correlated with an increased chance of correctly performing a cognitive task, suggesting that PAC improves the transfer of information in the brain (Tort et al., 2009, 2010; Fell and Axmacher, 2011; Kitchigina, 2018). In patients with AD-related dementia and mild cognitive impairment (MCI), a decreased theta-gamma PAC was observed when compared with healthy subjects, which worsened as the disease progressed (Goodman et al., 2018). Similar decreases of theta-gamma PAC have been observed in rodent models for AD (Goutagny et al., 2013; Gauthier-Umana et al., 2020). However, alterations in PAC during pre-plaque stages of AD have not been evaluated thoroughly.

Hippocampal function and neuronal activity are heavily dependent on brain states, which are strongly linked to different types of behavior (Fontanini and Katz, 2008; McCormick et al., 2020). Besides the brain states during sleep, several brain states can be defined based on the level of arousal, attention and movement, such as locomotor/exploration and wake immobile (Kay and Frank, 2019). Different neuronal circuits and hippocampal subcircuits are involved in these dynamic states which are tightly regulated by neuromodulatory systems (Buzsaki, 2006; Trimper et al., 2017). Changes in the activity of these neuromodulatory systems have been shown to affect oscillatory patterns during specific types of behaviors (Gordon et al., 2005; Gurevicius et al., 2013; Trimper et al., 2017). Investigating changes in oscillatory activity during behavioral states could therefore offer novel insights into underlying neuromodulatory disease mechanisms (Nimmrich et al., 2015). Neuromodulatory systems such as the noradrenergic system and cholinergic system have been demonstrated to be affected at early stages of AD, which could influence oscillatory hippocampal activity during different behavioral states (Braak and Braak, 1991; Dubelaar et al., 2006; Braak et al., 2011; Gurevicius et al., 2013; Byron et al., 2021; van den Berg et al., 2022).

Early synaptic dysfunction has been demonstrated to disrupt network activity. The majority of the hippocampal local field potential (LFP) animal studies were performed at relatively advanced stages, when Aβ were already present in the brain. However, sAβ species have been demonstrated to alter network activity before Aβ plaques are present in the brain, therefore, we hypothesized that early AD related pathological changes would interfere with hippocampal network function in the pre-plaque stage of AD (Shah et al., 2016; Ben-Nejma et al., 2019; van den Berg et al., 2022). Moreover, we hypothesized that this hippocampal dysfunction would be different between behavioral states, which might offer new insights into early neuropathological mechanisms underlying AD-related hippocampal network disruption. Therefore, hippocampal local field potential recordings were performed in the highly translational TgF344-AD rat model, while animals were exploring an open field.

## 2. Materials and methods

### 2.1. Animals and ethical statement

All procedures were in accordance with the guidelines approved by the European Ethics Committee (decree 2010/63/EU) and were approved by the Committee on Animal Care and Use at the University of Antwerp, Belgium (approval number: 2019–06). The animal model for AD used in this study was the TgF344-AD rat model, which bears the human APP gene with Swedish mutation and the human PS1 gene with the deltaE9 mutation, resulting in an age-dependent accumulation of human amyloid. Rats were bred inhouse by breeding heterozygous TgF344-AD rats with wild-type F344 rats (Charles River, Italy). The male offspring were used in this study. The TgF344-AD rats were heterozygous for the human transgenes while the DNA of the wildtype littermates did not bear the human transgenes. Electrophysiological experiments were performed in 4-month-old TgF344-AD rats (*N* = 5) and wild-type littermates (*N* = 5). Rats were group-housed prior to implantation but housed separately afterward. All animals were kept on a reversed, 12 h light/dark cycle, with controlled temperature (20–24°C) and humidity (40–60%) conditions. Standard food and water were provided *ad libitum*.

### 2.2. Chronic hippocampal electrophysiological measurements

#### 2.2.1. Surgical procedure

Anesthesia was induced using 5% isoflurane (Isoflo^®^, Abbott Laboratories; medical air 1 L/min) and maintained using 2–3% isoflurane (at 1 L/min) during the surgery. Animals were placed in a stereotaxic frame and a craniotomy was made above the right dorsal hippocampus (AP-3.00, ML 2.50). A 16-channel laminar electrode (E16 + R-100-S1-L6 NT, Atlas Neuro-engineering, Belgium) with internal reference was carefully lowered (DV 2.5–3.5 mm) into the dorsal hippocampus. The pointy tip feature of the electrode allows penetration of the dura without the need to open or remove the dura. The exact depth of the recording sites was identified online by the layer-specific local field potentials (LFP) of the hippocampus. The craniotomy was sealed with a sterile silicone gel (Kwik-Cast, WPI). Stainless steel screws were drilled into the skull overlaying the olfactory bulb, frontal cortex, left hippocampus, and cerebellum, of which the latter served as ground electrode. The implant was covered in several layers of dental cement (Stoelting) and the wound was closed. Rats were treated with antibiotics until 3 days after the surgery (5 mg/kg, Enrofloxacin (Baytril®, Bayer) (in drinking water) and analgesics were administered (0.05 mg/kg Buprenorphine (Temgesic®, Indivior Europe Limited) subcutaneous during surgery, followed by 2 daily injections of 5 mg/kg Carprofen (Rimadil®, Pfizer) for 2 days after surgery. Rats were allowed to recover for at least 7 days prior to the LFP recordings.

#### 2.2.2. Neurophysiological data acquisition

The electrophysiological recordings were acquired during the open field test (OFT) when the animals were 4.5 months old. The open field setup consisted of a square box (1 m × 1 m), a camera mounted to the ceiling that captured the complete open field (sampling rate camera: 30 frames per second), and visual cues on the walls to assist spatial orientation. The animals were placed in the open field, without prior habituation, for two consecutive days for the duration of 1 h on the first day (trial 1) and 20 min on the second day (trial 2). Rewards were used to promote explorative behavior when the animals were in the box. Hippocampal LFP’s were recorded using a wireless, 16-channel headstage (sampling rate 25 kHz, W2100 system, Multichannel Systems, Germany) and animal movement was tracked using Anymaze soft-and hardware (Anymaze, Stoelting, Dublin). TTL pulses were used to synchronize the video recording and LFP acquisitions.

#### 2.2.3. Tissue collection validation of the electrode position

Anesthesia was induced with 5% isoflurane and was maintained at 2–2.5%. An electrical current (30 μA, 3 s) was applied *via* the electrode at the top, middle, and bottom channels to allow validation of the electrode position. Thereafter, the animals were euthanized *via* intravenous injection of 50 mg/kg pentobarbital (Dolethal ®, Vetoquinol, Belgium), followed by cardiac perfusion with ice-cold phosphate-buffered saline (PBS) and 4% Paraformaldehyde (Merck Millipore, Merck KGaA, Darmstadt, Germany). The brains were surgically removed and postfixed for 4–6 h using 4% PFA. A sucrose gradient was applied (5, 10, and 20% Sucrose in 0.1 M PBS), after which the brains were snap frozen using liquid nitrogen and stored at –80°C until further processing.

The frozen brains of the animals were sliced into 12 μm thick coronal sections using a cryostat (Cryostar NX 70, Thermo Fisher Scientific). The sections were stained with Nissl staining (Cresyl Violet 0.1%, Sigma-Aldrich) and studied under the light microscope to validate the position of the electrodes for each animal.

#### 2.2.4. Validation of the electrode position

**Figure 1:**
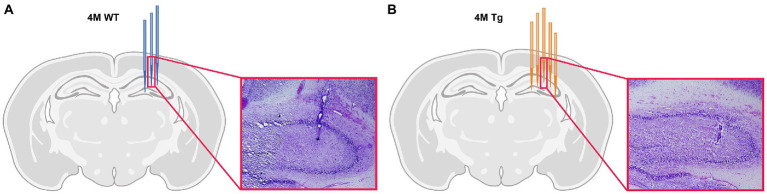
Histological validation of electrode position. **(A)** Schematic overview of electrode position in 4-month-old Wild type (WT) animals. Each blue electrode represents track of the electrode of a different WT subject. **(B)** Schematic overview of the electrode position in 4-month-old TgF344-AD (Tg) animals. Each orange electrode represents track of the electrode of a different Tg subject. The red insert shows exemplary light microscopy images of Nissl-stained brain slices showing the electrode trace in the hippocampus of a WT animal **(A)** and TgF344-AD rat **(B)**.

Each layer of the hippocampus has distinct functions and therefore, distinct oscillatory activity. The main focus of this study is on the different layers of the CA1 region. Therefore, histological electrode validation was performed. Based on the location of the tip of the electrode (Figure 1), we could infer which channels were placed in the different layers of CA1. These channel locations were further validated by the distinct oscillatory patterns of the different hippocampal layers, such as occurrence of sharp wave-ripple and theta power across different hippocampal layers. This channel information was used in further analyses. For 2 WT animals, validation was not possible because the lesions were not visible under the microscope and are not shown in the figure. However, a comparison of the distinct oscillatory patterns with other WT animals showed no differences. Therefore, they were still included in the data analysis.

#### 2.2.5. Analysis of electrophysiological and behavioral data

##### 2.2.5.1. Behavioral states and segmentation

Based on pre-defined speed thresholds originating from the animal tracking, we classified the behavior during each video frame into exploration (>0.05 m/s), grooming (0–0.05 m/s), and wake immobility (0 m/s) (Hoydal et al., 2007; Sharif et al., 2021). The main focus of this paper was to investigate how hippocampal activity is altered during exploration and wake immobility behavior, therefore, we excluded epochs which were scored as grooming for all further analyses (Buzsaki, 2015; Colgin, 2016). Moreover, epochs with a duration shorter than 1 s were removed from further behavioral and electrophysiological analysis. Video recordings were used to validate the classified behavior of the animals. The TTL pulses stored in the LFP data were used to synchronize the brain activity with the video frames, to facilitate segmentation of the LFP signals into the two behavioral states using Fieldtrip (Oostenveld et al., 2011).

##### 2.2.5.2. Power analysis of hippocampal LFPs

Power spectra were calculated using fast Fourier transform in Brainstorm (Tadel et al., 2011) for each behavior state using the Welch’s method (window size 1 s, 50% overlap). The sum of the total power between 0.5–250 Hz was calculated. Next, the power for both behavioral states was calculated for specific frequency bands of interest, i.e., delta (0.4–4 Hz), theta low (5–8 Hz), theta high (8–12 Hz), slow gamma (30–45 Hz), fast gamma (60–120 Hz) and high frequency oscillations (HFO) (120–250 Hz). The normalized power was calculated as the fraction of a specific frequency band power in the total power over all frequency bands to minimize variation in amplitude due to differences in the exact placement and properties of the electrode. Normalized power spectra were averaged across genotypes separately for the behavioral state. For each frequency band, the power across the three channels with the highest power was averaged for each subject and compared between groups, behavioral state, and trial.

##### 2.2.5.3. Phase-amplitude coupling analysis

The amplitude of the hippocampal gamma rhythm is modulated by the phase of theta, which is called phase-amplitude coupling (PAC). To evaluate the strength of the theta-gamma coupling, the modulation index (MI) was used, that after bandpass filtering uses the phase of the slow oscillation and the amplitude envelope of the fast oscillation to create a complex vector of which the length represents the amplitude of each fast oscillation whereas the phase of the slow oscillation is represented by the angle (Canolty et al., 2006; Tort et al., 2010). In the case of an absence of PAC, these vectors form a roughly uniform circular shape centered around zero, however if there is modulation, then the amplitude at a certain phase is higher, which will create a bump in the polar plot. The MI is derived from the mean vector length and represents the strength of the phase-amplitude coupling.

The analysis of the PAC was performed on one channel, which was placed in the pyramidal layer of the CA1, based on the electrode validation described in 2.2.4 (Figure 1), was used for the PAC analysis. First, for each subject, a comodulogram was calculated which demonstrates the MI for each pair of frequencies. The theta band (5–12 Hz) and gamma band (30–120 Hz) were divided into 1 Hz bins and for each combination of theta and gamma frequencies the mean modulation index across an epoch was calculated and averaged across epochs to create a subject based comodulogram. Next, a group-averaged comodulogram was computed in Matlab which demonstrated for each group the main frequencies of modulation. The main frequency modes of modulation were examined and were used to perform a time-resolved PAC (tPAC) analysis. tPAC computes the MI between specific frequency bands estimated from the group-averaged comodulogram (6.5–10 Hz vs. 55–90 and 6.5–9 Hz vs. 35–45 Hz during exploration and 6.5–9 Hz vs. 60–90 Hz during wake immobility) for each 1-s epoch (Samiee and Baillet, 2017). These modulation indices were averaged over time to create a subject averaged MI. These time-averaged modulation values were statistically compared between groups and trials.

##### 2.2.5.4. Offline detection and analysis of sharp wave-ripples

Analysis of sharp wave-ripple activity was performed on one channel which was placed in the CA1 layer of the hippocampus, based on the electrode validation as described in section 2.2.4. All preprocessing steps were conducted using the Fieldtrip toolbox while detection and analysis of the SWR were done with in-house MATLAB codes. First, to detect ripples in the pyramidal layer of CA1, the wide-band signal was band-pass filtered between 120 and 250 Hz using a 400^th^ order Butterworth infinite response filter and was afterwards down sampled to 1,200 Hz. Next, the filtered data was segmented based on the obtained ‘exploration’ and ‘wake immobility’ epochs. The segmented data was z-scored, rectified, and smoothed using a rectangular filter window with a length of 8 ms, generating the ripple power signal (Molle et al., 2006). SWR were identified in the top channels which were placed in the pyramidal layer of the CA1 when the ripple power exceeded the threshold of 3SD from the channel mean. Events were expanded until the power fell below 2SD, events with a duration shorter than 30 ms and/or a peak spectral frequency lower than 140 Hz were discarded. Afterward, a thresholding algorithm was applied to detect sharp waves in the stratum radiatum (Oliva et al., 2018). Signals in the channel which demonstrated strong sharp waves were bandpass filtered between 0.5 and 40 Hz using a 400th order Butterworth filter. Sharp wave events which lasted between 20 ms and 400 ms were detected when the power of the filtered signal exceeded the threshold of 2.5SD from the mean. Only ripples that co-occurred with SW were kept for further analysis. Power in the SWR band, peak spectral frequency, duration, and spectral frequency in the slow gamma range during the SWR were extracted and were statistically compared between groups, state, and trial.

To test if there was a significant genotypical difference in SWR occurrence between behavioral states. SWR occurrence is dependent on electrode placement and electrode properties. To limit this bias, a ratio between the occurrence of SWR during exploration and wake immobility was calculated and was statistically compared between groups and trial.

##### 2.2.5.5. Behavioral analysis

During the experiments in the OFT, the animals were tracked based on the center point of the body for the complete length of the trial *via* Anymaze. Based on this tracking, the total distance that each animal traveled in the open field was extracted for each trial. Secondly, during the open field test, the open field was divided into an outer and an inner area. This separation was used to extract the following parameters: the time each animal spent in the inner area, the total distance traveled in the area and the number of entrances to the inner area per animal were extracted. The different behavioral parameters were statistically compared to evaluate the effects of genotype and trial. Next, all trials were segmented into epochs of 5 min to evaluate if behavioral genotypical differences were observed during specific moments in the trial and the parameters were compared between groups, trial, and epoch.

##### 2.2.5.6. Statistics

The statistical analysis of the data was performed using the JMP Pro software (Version 16, SAS Institute Inc., Cary, NC, United States, 1989–2021). Regarding the analysis of the power of the hippocampal oscillations and SWR characteristics a linear mixed model analysis (LMM) was performed, to evaluate the main effects of genotypes, state and trial and the interactions (genotypes*state, genotypes*trial, state*trial, genotypes*state*trial) of these effects. First, outliers were detected per genotype, state, and trial, based on principal component analysis. Data points with a T2 statistics indices higher than the 95% confidence interval were excluded from the analysis. Next, random intercept models were constructed for each frequency band and/or SWR parameter, with genotype, state and trial and the interactions between these variables as fixed effects and the variable subject as a random effect. A *post hoc* test was performed if significant main effects and/or interaction effects were observed, using the Students T-test followed by a false discovery rate (FDR) correction using the Benjamini–Hochberg method with *p* < 0.05. The Benjamini–Hochberg procedures was used to calculate the *q*-value, which represents the FDR corrected value of *p*.

Regarding the analysis of the PAC, the occurrence rate ratio of SWRs and the analysis of the behavior of the rats across the entire trial, a LMM was used to evaluate the main effects of genotypes, trial and the interaction between genotypes and trial for each behavioral state. Outlier detection was again performed using a principal component analysis based on the T2 statistics as described earlier. Next, a random intercept model was generated for each behavioral state and frequency band of interest. Genotype, trial, and genotype*trial were used as fixed effects, and subjects was used as random effects. In the case when no significant genotype*trial interaction was present, the interaction was removed from the LMM, and the model was recalculated using genotype and trial and those *p* values are reported. In case of significant main effects and/or interaction effects were observed, Students *T*-test were performed, followed by a FDR correction using the Benjamini–Hochberg method with *p* < 0.05. The Benjamini–Hochberg procedure was used to calculate the *q*-value, which represents the FDR corrected value of *p*.

To evaluate if behavioral performance was altered during different stages of the trial, an additional statistical analysis was performed on the epoched data. A LMM with epoch, genotype and epoch*genotype as fixed effects and subject as random effect were used. The interaction term was removed from the model in case it was not significant, and the model was recalculated using genotype and trial as fixed effects, and those p values are reported. In case of significant main effects and/or interaction effects were observed, *post hoc* tests were performed as described above.

Graphical representation of the data was obtained u GraphPad Prism (version 9.2.0 for Windows, GraphPad Software, San Diego, CA, United States^1^).

## 3. Results

### 3.1. Altered power of hippocampal oscillations in TgF344-AD rats while exploring a novel environment

**Figure 2:**
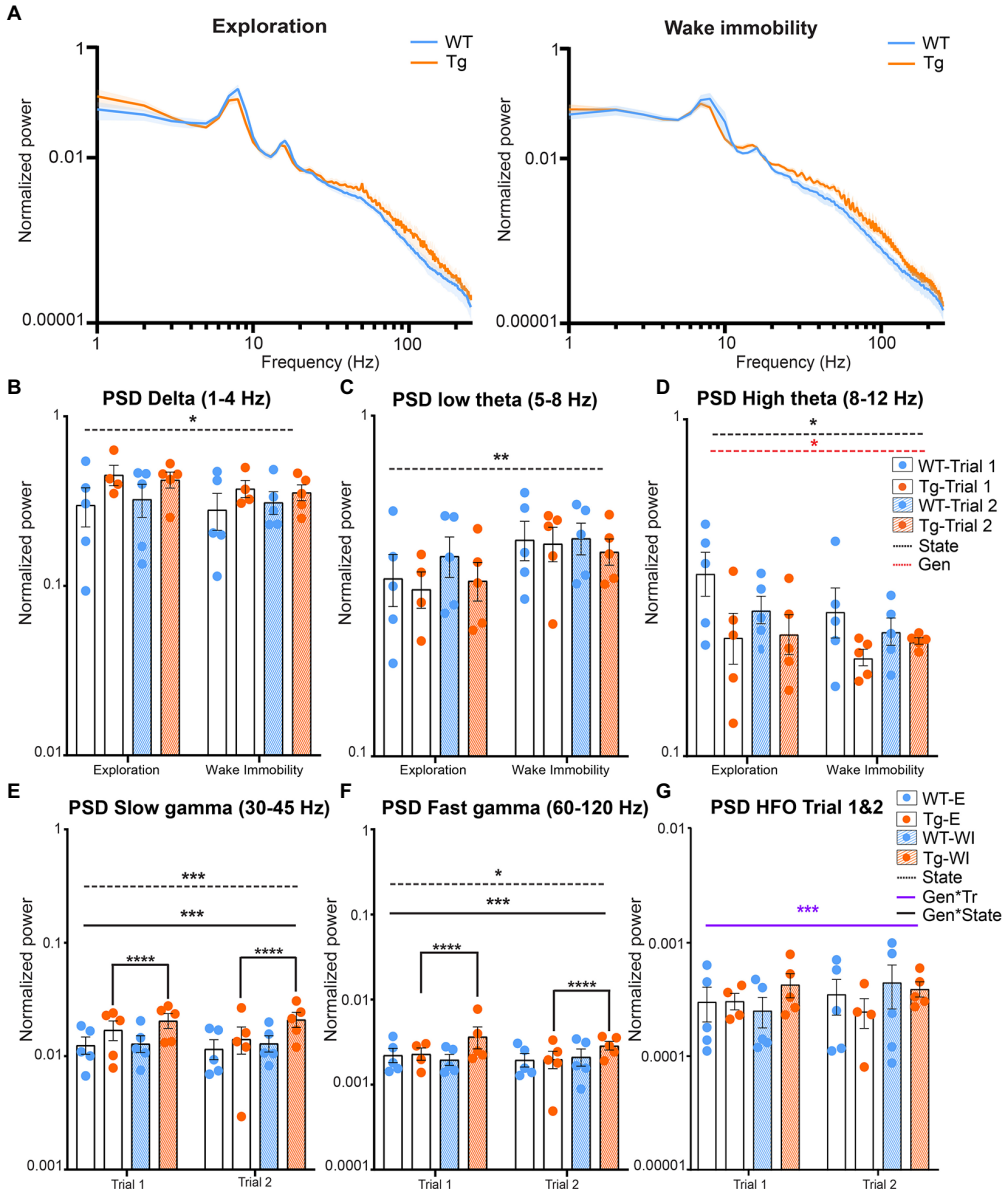
Power during explorative and wake immobility behavior in TgF344-AD rats and wildtype littermates. **(A)** Mean normalized power spectra during explorative behavior (left) and wake immobility (right) at trial 1. Graphs show the mean power across groups. Shading indicates the SEM across the groups. **(B)** Averaged normalized power (mean ± SEM) of the delta oscillations at trial 1 and trial 2 during exploration and wake immobility (WI). **(C)** Averaged normalized power (mean ± SEM) of the low theta oscillations at trial 1 and trial 2 during exploration and wake immobility (WI). **(D)** Averaged normalized power (mean ± SEM) of the high theta oscillations at trial 1 and trial 2 during exploration and wake immobility (WI). **(E)** Averaged normalized power (mean ± SEM) of slow gamma oscillations during exploration (E) and wake immobility (WI) across trials. **(F)** Averaged normalized power (mean ± SEM) of fast gamma oscillations during exploration (E) and wake immobility (WI) across trials. **(G)** Averaged normalized power (mean ± SEM) of high-frequency oscillations (HFO) during exploration (E) and wake immobility (WI) across trials. PSD, power spectral density, *p ≤ 0.05, **p ≤ 0.01, ***p ≤ 0.001, ****q ≤ 0.0001.

Aβ and pTau are known to interfere with synaptic function and thus are expected to induce altered oscillatory activity which is linked to the cognitive symptoms of AD (Busche et al., 2012; Ma and Klann, 2012; Ben-Nejma et al., 2019; Hector and Brouillette, 2020; Li and Selkoe, 2020). These alterations have been linked to altered spectral power at different frequencies of interest. To visually evaluate potential differences in the power of different neuronal oscillations between genotypes and different behavioral states, the mean power across all frequencies was calculated and statistically compared between genotypes, state, and trial (Supplementary Table S1). Figure 2 demonstrates the power spectra during wake immobility and exploration for both genotypes during trial one (Figure 2A). The power within certain frequency bands of interest was quantified and the results are shown in Figure 2B. We observed no significant trial effects in any of the classical frequency bands (δ, θ, and γ). However, for the high frequency oscillations (120–250 Hz) we did observe a significant trial effect (Figure 2C).

A significant behavioral state effect (*p* = 0.0281) was observed for the mean power of delta oscillations demonstrating an increased power during explorative behavior when compared with wake immobility behavior for both the TgF344-AD rats and their WT littermates (Figure 2B). A significant state effect was also observed for the mean power of low theta oscillations (*p* = 0.0035) demonstrating an increased power during wake immobility behavior when compared with explorative behavior for both genotypes (Figure 2C). A significant state (*p* = 0.0277) and genotype effect (*p* = 0.0474) were observed for the mean power of the high theta oscillations. The overall power in the high theta range was decreased in TgF344-AD rats compared to WT littermates. Moreover, high theta power during explorative behavior was found to be increased, which was mainly driven by the WT rats (Figure 2D).

Similarly, a significant genotype*state interaction (*p* = 0.009) was observed for the mean power of the slow gamma oscillations. *Post hoc* analysis demonstrated an increased power during wake immobility behavior when compared with explorative behavior for the TgF344-AD rats (*q* = 0.000025, Figure 2E) but not in WT rats (*q* = 0.4235). Moreover, no significant differences in slow gamma power were observed between genotype during both stages (Supplementary Table S2).

Similarly, a significant genotype*state effect was also observed (*p* = 0.0046) for the mean power of the fast gamma oscillations. Post-hoc analysis demonstrated an increased power during explorative behavior when compared with wake immobility behavior for the TgF344-AD rats (*q* = 0.0021, Figure 2F and Supplementary Table S2).

Lastly, a significant genotype*trial interaction (*p* = 0.0191) was observed for the mean power of the high frequency oscillations, range [120–250 Hz]. However, *post hoc* analysis demonstrated no significant difference between genotypes or trials (Figure 2G and Supplementary Table S3).

### 3.2. Decreased modulation of gamma amplitude in TgF344-AD rats

**Figure 3:**
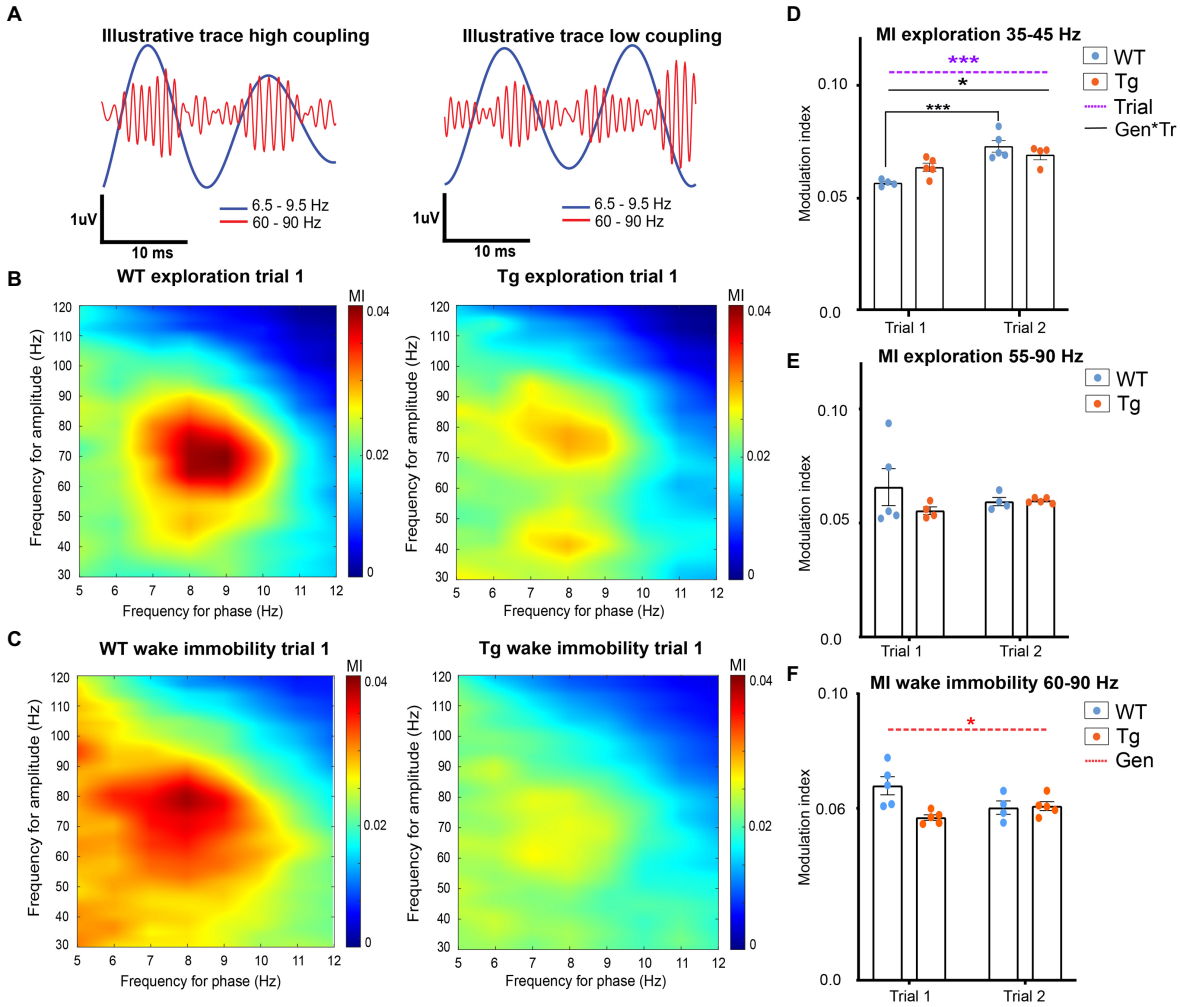
Hippocampal theta-gamma coupling in TgF344-AD rats during exploration and wake immobility. **(A)** Illustrative filtered traces (theta range in blue, gamma range in orange) of a respective wildtype (WT) and TgF344-AD (Tg) subject during exploration. The amplitude of the gamma oscillations in the left trace demonstrated high coupling to the phase of theta, whereas the amplitude of the gamma oscillations is less coupled to the theta phase. Average comodulograms demonstrate main frequencies of modulation during exploration **(B)** and wake immobility **(C)** in WT rats (left) and Tg rats (right). Colors indicate the strength of the PAC as calculated by the modulation index (MI) for different frequencies of theta (*x*-axis, phase-determining frequency) and gamma (*y*-axis, frequency for amplitude) (paragraph 2.2.5.3). Comodulograms demonstrate coupling between 6.5–9 Hz and 35–45 Hz, and 6.5–9 Hz and 55–90 Hz during exploration. During wake immobility, coupling between 6.5–9 Hz and 60–90 Hz were observed. These results were used to investigate the coupling strength for each 1-s epoch between these specific frequency bands, to obtain subject averaged time-resolved MI. **(D–F)** time resolved PAC analysis results (paragraph 2.2.5.3). **(D)** Time-resolved averaged MI between theta (6.5–9 Hz) and gamma frequencies (35–45 Hz) during exploration. **(E)** Time-resolved averaged MI between theta (6.5–9.5 Hz) and gamma frequencies (60–90 Hz) during wake immobility. **(F)** Time-resolved averaged MI between theta (6.5–9.5 Hz) and gamma frequencies (60–90 Hz) during wake immobility Bars indicate mean MI (±SEM), dots represent subject values. *p ≤ 0.05, **p ≤ 0.01, ***p ≤ 0.001/*q* ≤ 0.001.

Phase-amplitude coupling (PAC) is an electrophysiological phenomenon where the amplitude of the fast oscillations (gamma oscillations) is modulated by the phase of the slower oscillations (theta oscillations) (Figure 3A). The theta-gamma PAC has an important function in both memory encoding and memory retrieval, functions which are known to be affected in AD (Witton et al., 2016; Bergmann and Born, 2018).

In order to calculate PAC, the main frequencies by which theta frequencies modulate the amplitude of gamma oscillations were analyzed by calculating a group-average comodulogram as described previously (Tort et al.). The theta band (5–12 Hz) and gamma band (30–120 Hz) were divided into 1 Hz bins and for each combination of theta and gamma frequencies the mean modulation index across all epochs from each subject was calculated to create a subject based comodulogram for each trial, which was averaged to create group-averaged comodulograms. In WT, comodulograms demonstrated coupling between theta frequency 6.5–10 Hz and fast gamma 55–90 Hz during exploration (Figure 3B) and between theta frequency 6.5–9 Hz and fast gamma 60–90 Hz during wake immobility (Figure 3C). The comodulogram of the TgF344-AD rats demonstrated similar peaks, as well as a second coupling between theta frequency 6.5–9 Hz and slow gamma 35–45 Hz during exploration (Figures 3B,C).

Next, to evaluate the strength of the PAC, a time-resolved PAC analysis was performed. For each of the frequency bands of interest according to the comodulograms a separate tPAC analysis was performed. For each 1-s-long segment of data, the MI between target theta and gamma oscillations was calculated and averaged across epochs, to create a subject-based MI over time (paragraph 2.2.5.3). These subject-averages were statistically compared between groups and trials.

During exploration, no significant interaction, nor genotype or trial effects were observed in the coupling between fast gamma (55–90 Hz) and theta (6.5–10 Hz), demonstrating that the PAC during exploration is not different between 4-month-old TgF344-AD rats and WT littermates (Figure 3E and Supplementary Table S4). Statistical analysis of the theta-gamma coupling strength between slow gamma (35–45 Hz) and theta (6.5–9 Hz) demonstrated a significant trial effect (*p* = 0.0003) demonstrating an increased coupling during trial 2, and a significant genotype*state interaction effect (*p* = 0.0181) (Figure 3F). *Post hoc* analysis reveals a significant increase in coupling strength between trials in WT (*q* = 0.0009), but not in TgF344-AD rats (Figure 3D and Supplementary Table S5). Statistical analysis of the PAC between theta and fast gamma during wake immobility demonstrated a significant genotype effect (*p* = 0.0284) (Figure 3F). This showed that the MI in TgF344-AD rats were significantly lower during wake immobility compared to WT littermates irrespective of trial, however, when looking at the graph, this decrease seems more prominent in trial 1.

### 3.3. Altered SWR activity in TgF344-AD rats while exploring novel environment

Sharp wave-ripple are an important feature of memory function and are thought to represent a major factor in online memory consolidation and offline memory replay. SWRs are present both during exploration and wake immobility and are the result of the delicate interplay between inhibitory and excitatory neurons in the CA1 layer. The slow gamma oscillations observed during SWR are a phenomenon characteristic for SWR during waking and these oscillations have been associated with synchrony between the CA3 and CA1 layer. This synchronization is hypothesized to enable coordinated memory reactivation in the hippocampus, a process that might be disrupted in AD (Carr et al., 2012; Gillespie et al., 2016; Sanchez-Aguilera and Quintanilla, 2021).

**Figure 4:**
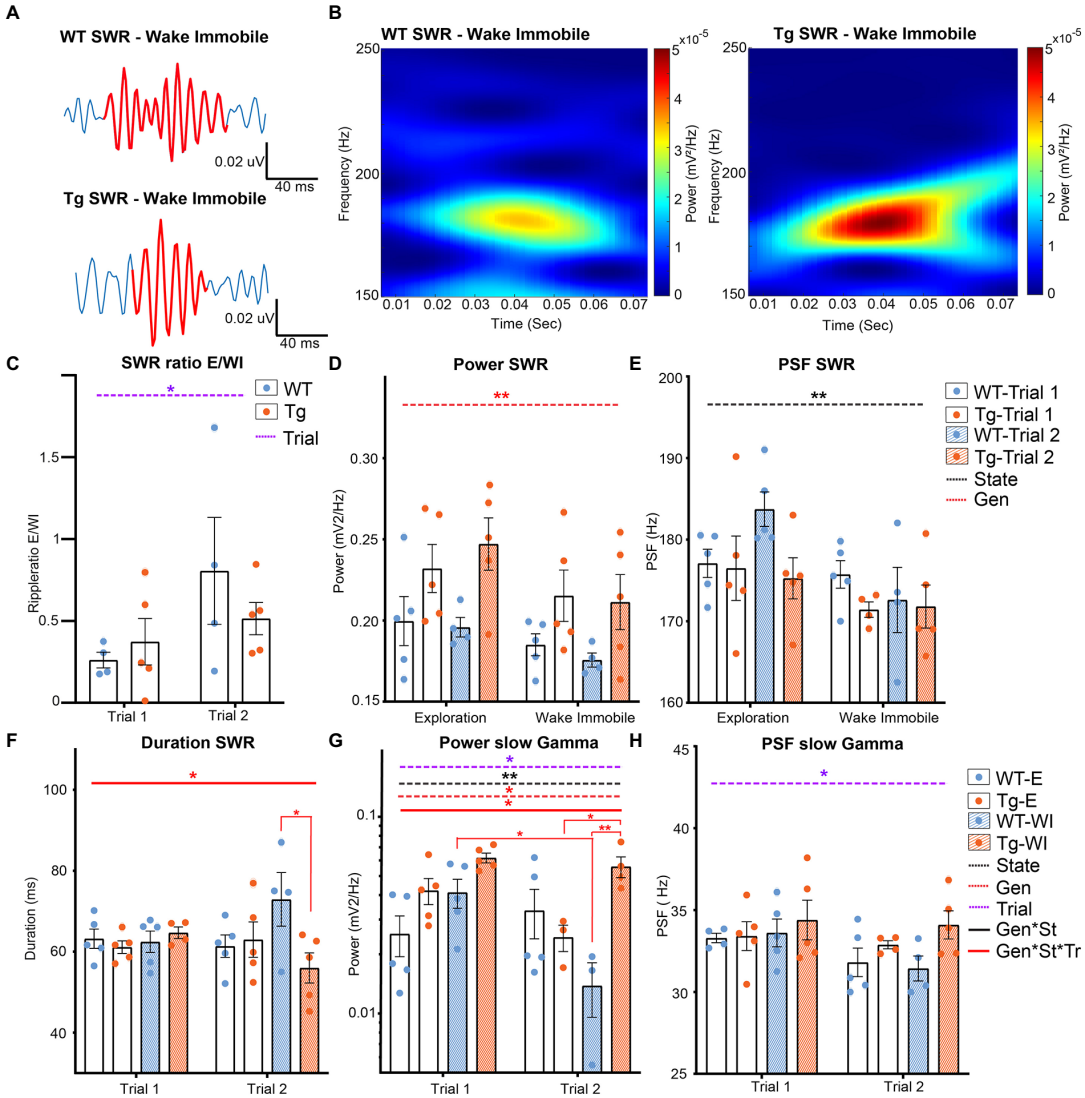
Analysis of different parameters for SWR in TgF344-AD rats during exploration and wake immobility. **(A)** Illustrative traces of bandpass filtered SWR (120–250 Hz) during wake immobility and corresponding time-frequency plot **(B)** demonstrating the power of the ripple oscillation at different frequencies over time for the wildtype (WT) and TgF344-AD (Tg) rat. **(C)** SWR occurrences ratio between exploratory/wake immobility for each animal. **(D)** The mean power within the SWR frequency band for each behavioral state. **(E)** Peak spectral frequency of the SWR for each behavioral state. **(F)** Duration of SWR in milliseconds (ms) separated for each trial. **(G)** The power of slow gamma oscillations during SWR was separated for each trial. **(H)** The peak spectral frequency of slow gamma during exploration and wake immobility separated for each trial. Bars represent mean ± SEM, individual values are represented by the dots. *p ≤ 0.05/*q* ≤ 0.01, **p ≤ 0.01/*q* ≤ 0.01.

After SWR extraction, SWR occurrence rates, peak spectral frequency (PSF) of SWR and slow gamma oscillations, the power of SWRs and slow gamma oscillations and the duration of the ripples was statistically compared using LMM (Supplementary Table S6 and Figure 4). Analysis of the SWR occurrence rates showed a significant trial effect (*p* = 0.0342), demonstrating an increased occurrence rate of SWR during trial 2 compared with trial 1 (Supplementary Table S7 and Figure 4C).

A significant genotype effect was observed in the power at SWR frequencies (*p* = 0.0013), showing a significantly higher power in TgF344-AD rats compared to WT littermates (Figure 4D). The PSF of the SWR frequencies showed a significant state effect (*p* = 0.002) demonstrating a higher PSF_SWR_ during exploration compared to wake immobility (Figure 4E). Studies have demonstrated that the duration of SWRs is altered in animal models of AD (Sanchez-Aguilera and Quintanilla, 2021). Analysis of the duration of the SWR in TgF344-AD rats demonstrated a significant interaction effect (genotype*state*trial *p* = 0.0286). *Post hoc* analysis shows a significant decreased duration of SWR during wake immobility in TgF344-AD rats during trial 2 (*q* = 0.0252), but not during trial 1 (Supplementary Table S8 and Figure 4F).

Analysis of the slow gamma frequencies showed a significant genotype (*p* = 0.0177), trial (*p* = 0.0082) and state effect (*p* = 0.0229) in the power of the slow gamma oscillations, together with a significant interaction between those parameters (*p* = 0.046). Post-hoc analysis revealed a significant decrease in power in WT rats during wake immobility between trial 1 and trial 2 (*q* = 0.0488, Figure 4G and Supplementary Table S8). In addition, a significant difference in power was observed between WT and TgF344-AD rats during wake immobility in trial 2, but not during trial 1 (*q* = 0.0096). Moreover, a significantly higher power during wake immobility with respect to exploration was observed in the TgF344-AD rats in trial 2 (*q* = 0.0496). A significant trial effect (*p* = 0.0346) was observed in the PSF at the slow gamma frequencies demonstrating a significantly higher PSF during trial 1 compared to trial 2 (Figure 4H).

### 3.4. Alterations in oscillatory activity concur with measures of increased anxiety

**Figure 5:**
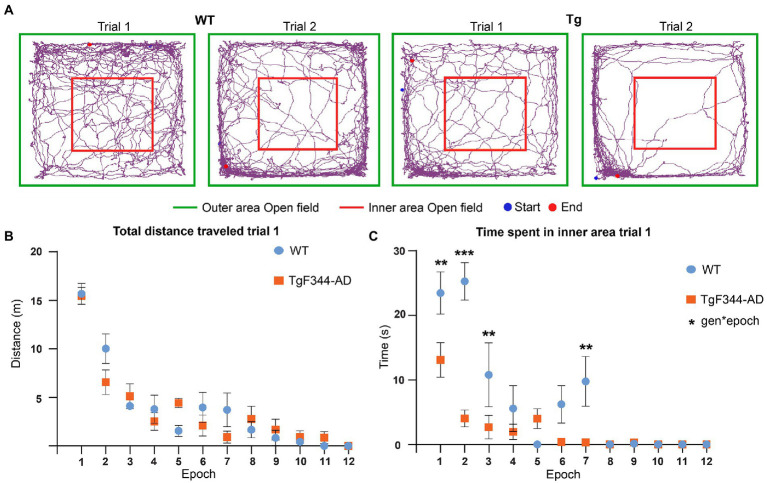
Behavioral analysis OFT during trial 1. **(A)** Movement traces of representative wild-type (WT) (left) and TgF344-AD (Tg) (right) rat during trial 1 and trial 2. The start position is indicated with the blue dot and the end position is indicated with the red dot. The 1 × 1 m open field is divided into an inner area (red) and outer area (green). **(B)** Total distance traveled in meters across different 5-min epochs during trial 1. **(C)** Time spent in seconds in the inner area of the open field across different 5-min epochs during trial. Plots represent mean ± SEM. **q ≤ 0.01, ***q ≤ 0.001.

Previous studies in TgF344-AD rats observed increased anxiety in the open field test from 4 months onward. Behavioral analysis was performed to validate these findings in our TgF344-AD rats. Increased anxiety in rats in the open field is characterized by spending more time in the outer area and less motor activity during exploration (Figure 5A). Total distance traveled, total distance traveled in the inner area, time spent in the inner area, and the number of entries to the inner area of the open field were extracted and statistically compared between trials and genotype. A significant genotype effect was observed in the time spent in the inner area, showing a significantly decreased time spent in the inner area in TgF344-AD rats during both trials (*p* = 0.018) (Supplementary Figure S1 and Supplementary Table S10). For all parameters, a significant trial effect was observed (Supplementary Figure S1 and Supplementary Table S10), demonstrating decreases in total distance traveled, distance traveled in the inner area, time spent in the inner area, and number of entries in the inner area during the second trial. Therefore, a second analysis was performed separately for each trial, to evaluate if genotypical differences were present during specific epochs within each trial. Trials were segmented into epochs of 5 min and statistically compared. Statistical analysis showed significant main epoch effects for all parameters during both trials demonstrating decreased explorative behavior over the duration of each trial (Supplementary Tables S11, S14).

No significant genotype or interaction effects were observed in the total distance traveled, suggesting that explorative behavior was not altered in TgF344-AD rats (Figure 5 and Supplementary Tables S10, S11, S14). However, a significant genotype (*p* = 0.0001) and interaction effect (*p* = 0.0001) was observed in the time spent in the inner area. TgF344-AD rats spent less time in the inner area during several epochs, mainly during the first half of the trial (Figure 5C and Supplementary Tables S10, S11). Moreover, a significant interaction effect was observed in the distance traveled in the center. During the second epoch, the TgF344-AD demonstrated a decreased distance traveled in the inner area (Supplementary Figure S2 and Supplementary Tables S11, S12). No significant genotype or interaction effects were observed in the number of entries into the inner area (Supplementary Table S10).

During trial 2, significant epoch effects were again observed in all aforementioned parameters, showing decreased locomotion and exploration over the duration of the trial in both groups (Supplementary Figure S3 and Supplementary Table S14). Secondly, a significant genotype effect (*p* = 0.0263) was observed for the number of entries to the inner area, showing a decreased number of entries to the inner area for the TgF344-AD rats (Supplementary Figure S3F and Supplementary Table S14). No significant genotype effects or interaction effects were observed in the other behavioral parameters during trial 2. The aforementioned results suggest increased anxiety was present in TgF344-AD rats, mainly during trial 1.

## 4. Discussion

This study aimed to investigate if hippocampal network activity is altered during explorative behavior at the pre-plaque stage of AD, using hippocampal electrophysiology in freely moving TgF344-AD rats and WT littermates. We observed a significantly decreased power in high theta oscillations in TgF344-AD rats. In addition, a decreased strength of PAC, an important electrophysiological mechanism underlying hippocampal dependent memory processes, was observed in TgF344-AD rats during wake immobility. SWR analysis revealed no significant differences in ripple ratios between genotypes, however, significantly increased power of SWR was observed in TgF344-AD rats, together with a significant decrease in the duration of the ripples. Moreover, significant genotype, trial and state effects were observed in the power of slow gamma oscillations during SWR, which is thought to represent the interaction between the CA3 and CA1 regions of the hippocampus. The aforementioned results demonstrate that hippocampal activity in TgF344-AD rats is already altered before amyloid plaques are present in the brain, mainly while the animals were in the wake immobility state. These differences in oscillatory activity concurred with increased anxiety behavior in TgF344-AD rats, in the absence of differences in locomotion.

### 4.1. AD pathology In TgF344-AD rats

TgF344-AD rats display progressive Aβ plaque accumulation in the cortex and hippocampus starting from 6 months of age (Cohen et al., 2013). Studies recently demonstrated that at 4 months of age, increased concentrations of soluble Aβ monomers and oligomers were present in the brains of TgF344-AD rats, in the absence of cortical and hippocampal Aβ plaques (Sare et al., 2020; Ratner et al., 2021; van den Berg et al., 2022). In addition, TgF344-AD rats display p-tau accumulation in the locus coeruleus from 6 months onwards (Rorabaugh et al., 2017). However, no studies yet have examined if pTau accumulations are present at 4 months of age. Impaired spatial memory in the Barnes maze has been observed in TgF344-AD rats starting from 4 months of age, suggesting hippocampal impairments are already present during the pre-plaque stage of AD as is observed in this study (Fowler et al., 2022). In addition, working memory impairments and delayed memory retention have been observed in 6-month-old female TgF344-AD rats (Bernaud et al., 2022). Moreover, cholinergic dysfunction and neuroinflammatory responses have been observed from 6 months onward in TgF344-AD rats (Chaney et al., 2021). These results highlight the diverse phenotype of the TgF344-AD rats which highly resembles human AD.

The majority of the electrophysiological studies in animal models for AD pathology have been done in the late phases of AD. Electrophysiological studies observed alterations in oscillatory activity in the hippocampus of TgF344-AD rats from 9 months onward (Bazzigaluppi et al., 2018; Stoiljkovic et al., 2019). To our knowledge, this study is one of the few electrophysiological studies performed at the pre-plaque stage in TgF344-AD rats. Several studies have investigated alterations in hippocampal synaptic function and/or network function in TgF344-AD rats using electrophysiological measurements. *In vitro* investigation of the tri-synaptic circuit in the hippocampus demonstrated increased long-term potentiation and hyperexcitability in dentate granule cells in 6-month-old male TgF344-AD rats, which was shown to be driven by increased noradrenergic postsynaptic signaling (Smith and McMahon, 2018; Goodman et al., 2021; Smith et al., 2022). Interestingly, a recent MRI study we performed did demonstrate alterations in whole brain network activity in 4-month-old TgF344-AD rats, suggesting that the presence of soluble Aβ species interferes with network function already before Aβ plaques are present in the brain of TgF344-AD rats (van den Berg et al., 2022).

Many efforts were made from different research groups to characterize the behavioral and cognitive alterations in TgF344-AD rats. Studies have demonstrated significantly increased anxiety in TgF344-AD rats from 4–5 months of age (Pentkowski et al., 2018), which persisted while the animals were aging (Tournier et al., 2021; Kelberman et al., 2022). A recent study demonstrated hyperexcitability in the basolateral amygdala, a central part of the fear circuitry, in 6-month-old and 12-month-old TgF344-AD rats which display increased anxiety compared to age-matched WT littermates (Hernandez et al., 2022). The aforementioned results demonstrate increased anxiety-like behavior in TgF344-AD rats starting from 4 months of age. Which is in accordance with the results from the current study, where a significant decrease in time spent in the inner area of the open field was observed in TgF344-AD rats in comparison with their WT littermates. Significant trial effects were observed in all behavioral parameters, which demonstrated decreased distance traveled and decreased time spent in inner area during the second trial. These results suggest that the TgF344-AD rats do recognize the OFT as a familiar environment during the second trial, hinting that spatial memory is still intact at the pre-plaque stage of AD (Giovannini et al., 2001; Sare et al., 2020).

### 4.2. Alterations in power of hippocampal oscillations in TgF344-AD rats

The frequency and amplitude of oscillations are heavily dependent on vigilance and behavior. In the current study we compared the power of brain oscillations during exploration and during wake immobility. Theta oscillations are present in the whole HC during active exploration and rapid-eye-movement (REM) sleep but also during memory encoding and retrieval (Colgin, 2016; Bauer et al., 2021). In animals, theta oscillations can be divided into two types, type I, or atropine insensitive theta (6–12 Hz) which is dominant during exploration and type II, or atropine sensitive theta (4–9 Hz), which is dominant during wake immobility (Kramis et al., 1975; Buzsaki, 2002; Gu and Yakel, 2022). In the current study we observed for both groups these expected state effects for low theta (type II) and high theta (type I). In addition, a decreased power of type I theta oscillations was observed in 4-month-old TgF344-AD rats. These results are in concordance with previous studies which observed a decreased theta power in 9-month old TgF344-AD rats (Stoiljkovic et al., 2019). The power of theta oscillations in people suffering from AD is heavily dependent on the disease stage, with increases in theta power observed in MCI patients and patients suffering from AD related dementia (Musaeus et al., 2018; Jafari et al., 2020; Byron et al., 2021; Spinelli et al., 2022). However, only a limited number of animal studies have investigated alterations in theta power during presymptomatic stages of AD. Injection of amyloid 1–40 monomers has been shown to induce decreases in hippocampal theta power (Colom et al., 2010), whereas amyloid plaques have been associated with increases in cortical theta power (Jafari et al., 2020; Spinelli et al., 2022). Decreased theta power has been observed in several animal models of AD, even in the absence of Aβ plaques. These decreases in theta power have been correlated to memory deficits and are thought to be caused by functional impairment of GABAergic septal hippocampal neurons by soluble Aβ (Caravaglios et al., 2010; Villette et al., 2010; Scott et al., 2012; Nimmrich et al., 2015; Wang et al., 2017). Both type I and type II theta oscillations are also directly influenced by cholinergic neurons of medial septum (Bauer et al., 2021; Gu and Yakel, 2022). This could in part be responsible for the observed decreased theta since the cholinergic neurons in nuclei of the basal forebrain have been shown to be susceptible to AD pathology (Grothe et al., 2014; Brueggen et al., 2015; Jeong et al., 2016; Fernandez-Cabello et al., 2020; Herdick et al., 2020).

The current study observed a significant increase in power of both slow and fast gamma oscillations in TgF344-AD rats during wake immobility compared to exploration, an effect which was not observed in WT littermates. In healthy subjects and animals, an increase in the power of slow and fast gamma oscillations is observed during memory encoding, sensory processing and quiet wakefulness, similar to what is observed in the current study (Bragin et al., 1995). This increase in gamma power appears to predict if memory formation will be successful in humans but also in mice (Sederberg et al., 2007; Matsumoto et al., 2013; Palop and Mucke, 2016). However, several studies have demonstrated that neuronal hyperactivity can be observed at the prodromal stages of AD, which can lead to an increased power of slow and fast gamma oscillations (Hector and Brouillette, 2020). This neuronal hyperactivity perturbs hippocampal network function. Some studies have suggested that the increased gamma power during early stages of AD could be the result of compensatory mechanisms (Mondadori et al., 2006; Gaubert et al., 2019; Morrone et al., 2022). Moreover, a number of studies have reported conflicting observations regarding gamma oscillations. These divergent findings are thought to be caused by methodological differences (Nimmrich et al., 2015; Byron et al., 2021). In the current study we did not observe genotype effects in gamma oscillations at the pre-plaque stage in TgF344-AD rats.

### 4.3. Phase-amplitude coupling

Theta-gamma PAC can be found in different species including mice, rats, and humans. Research has shown that the theta-gamma PAC plays an important role in the execution of cognitive functions and might be a specific measure of working memory functioning in humans (Canolty and Knight, 2010; Goodman et al., 2018). The modulation of different gamma bands, namely slow gamma (30–60 Hz) and fast gamma (60–130 Hz) appears to be linked to different memory processes. Research has demonstrated that the coupling between theta phase and slow gamma oscillations is increased after successful retrieval of memories in humans (Mormann et al., 2005) and rodents (Tort et al., 2009; Igarashi et al., 2014; Tamura et al., 2017). Synchronization of CA1 with the inputs from CA3 by the slow gamma suggest that the coupling between theta and slow gamma is dominantly involved by memory retrieval rather than memory encoding. The PAC between theta and high gamma (60–130 Hz) is thought to be important for sensory processing and memory encoding (Belluscio et al., 2012; Newman et al., 2013; Bieri et al., 2014). Electrophysiological experiments in the hippocampus of healthy rats, and in the non-epileptogenic hippocampus of patients have shown that the strength of the theta-gamma PAC will increase during learning (Tort et al., 2008, 2009; Kitchigina, 2018). It seems that the theta-gamma PAC strength is correlated directly with the increase in correctly performing a cognitive task and that the temporal coordination of neural spikes by theta-gamma PAC improves the transfer of information in the brain (Tort et al., 2009; Fell and Axmacher, 2011; Kitchigina, 2018). Moreover, increased PAC between low theta oscillations and both low and fast gamma oscillations in the HC during memory retrieval is also correlated with (memory) task performance (Vivekananda et al., 2021). Research also showed that in humans, fast and slow gamma oscillations are linked to different phases of the low theta oscillations which correspond with findings in animal studies (Colgin et al., 2009; Vivekananda et al., 2021). Thus, theta-gamma PAC might have an important role in memory processes in the HC.

Different preclinical and clinical studies in animal models for AD and in patients with AD have demonstrated that a decreased theta-gamma PAC can be observed (Goutagny et al., 2013; Bazzigaluppi et al., 2018; Goodman et al., 2018; Kitchigina, 2018), similar to the observations in the current study. Interestingly, impairment of theta-gamma PAC was observed before the onset of Aβ plaque formation in the APP23 mice and in (Aβ overexpressing) TgCRND8 mice (Goutagny et al., 2013; Kitchigina, 2018). Moreover, a study in TgF344-AD rats observed decreased cortical PAC in 9-month-old TgF344-AD rats, which coincided with cerebrovascular dysfunction and vascular remodeling, suggestive of a role for neurovascular dysfunction in PAC (Joo et al., 2017). The current study observed decreased PAC in fast gamma frequencies during exploration, suggesting that sensory processing and memory encoding might be impaired in TgF344-AD rats at the pre-plaque stage. In WT rats, an increased MI in slow gamma coupling was observed between trial 1 and trial 2, which was absent in TgF344-AD rats. Since slow gamma oscillations have been linked to memory retrieval, these results might suggest that the memory retrieval is impaired in TgF344-AD rats. However, future analyses which record hippocampal activity during cognitive tasks could give novel insights into alterations in memory retrieval and memory encoding during pre-plaque stages of AD. The disease mechanisms underlying alterations in PAC are still elusive, however, studies have observed that the changes in PAC coincided with interneuron dysfunction (Stoiljkovic et al., 2015, 2019; Park et al., 2020) alterations in cholinergic signaling in the hippocampus (Stoiljkovic et al., 2015) and accumulation of pTau and (soluble) Aβ (Bazzigaluppi et al., 2018; Stoiljkovic et al., 2018; Etter et al., 2019).

### 4.4. Sharp wave-ripples

Memory consolidation is primarily associated with sleep while memory retrieval (or replay) is associated with waking behavior. Awake memory replay is mainly seen during brief periods of wake immobility, which will be enhanced during both novel and rewarding behaviors (Carr et al., 2011). SWR are irregularly occurring LFP patterns that are observed during immobility, slow wave sleep and consummatory behavior. SWR are the hallmark of memory replay and consolidation, as during SWR a large population of HC neurons is activated, often in sequences that recapitulate past or potential future experiences (Buzsaki, 2015; Jones et al., 2019). During exploration, the HC shows a high presence of theta oscillations (7–14 Hz) and during wake immobility theta oscillations will be replaced by SWR to process information obtained during exploration (Girardeau and Zugaro, 2011). This is also reflected in the results of the current study since the ripple ratio shows higher ripple occurrence during wake immobility compared to exploration.

We observed increased slow gamma power, SWR power and decreased duration of SWR in TgF344-AD rats at pre-plaque stage of AD. Similar decreases in the duration of SWR have been observed in several animal models for AD (Sanchez-Aguilera and Quintanilla, 2021; Zhen et al., 2021). Interestingly, an *in vitro* study on hippocampal slices of a mouse model of AD observed a similarly increased slow gamma power, SWR power and decreased duration, which was caused by dysfunction of parvalbumin positive interneurons (Caccavano et al., 2020). Research has shown that altered spike-timing and phase-locking of spiking during SWRs will interfere with the induction of synaptic plasticity (Sadowski et al., 2016; Cuestas Torres and Cardenas, 2020). Neuronal hyperactivity associated with early stages of AD, such as interneuron dysfunction as is observed in other animal models of AD, could interfere with the spike-locking during SWRs and therefore shorten the duration of the SWR (English et al., 2014; Fernandez-Ruiz et al., 2019; Caccavano et al., 2020). Input from the CA3 region is also an important modulator of SWR activity and gamma activity. *In vitro* studies have observed that SWRs and gamma oscillations can be directly modulated by modulating the activity of excitatory pyramidal neurons of the CA3 layer (Geschwill et al., 2020). In addition, the medial entorhinal cortex (mEnt) is the major input of somatosensory information to the hippocampus (Axmacher et al., 2008, Igarashi et al., 2014). Disruption of entorhinal signaling has been demonstrated to decrease SWR duration during quiet wakefulness (Yamamoto and Tonegawa, 2017; Oliva et al., 2018). The mEnt is a region affected early by (soluble) Aβ pathology and *in vitro* electrophysiological studies in TgF344-AD rats at 6 months of age has demonstrated hyperexcitability in the synapses between the mEnt and dentate gyrus (Smith and McMahon, 2018; Smith et al., 2022). The altered SWR activity therefore might be the result of network disturbances caused by the mEnt, suggesting that entorhinal signaling might be altered already during the pre-plaque stage of AD in TgF344-AD rats.

The power of slow gamma oscillations increases throughout the whole hippocampus during SWR (25–55 Hz), which is thought to be associated with the quality of spatial memory replay (Carr et al., 2012; Gillespie et al., 2016; Oliva et al., 2018). Previous studies have observed decreased slow gamma oscillations during SWR in several mouse models of AD (Gillespie et al., 2016; Iaccarino et al., 2018; Stoiljkovic et al., 2019). This decreased slow gamma power has been linked to Aβ induced interneuron dysfunction (Gillespie et al., 2016; Caccavano et al., 2020). However, these studies were performed at late stages of AD, when Aβ plaque pathology was extensive, which could explain the contradiction with the outcomes from the current study. A recent study observed increased gamma power in mild Aβ pathology, which declined as the disease progressed, suggesting an initial increase in gamma power during early stages of AD, similar to what is observed in the current study (Gaubert et al., 2019).

### 4.5. Limitations

The focus of this study was solely on alterations in the hippocampal oscillations. However, it is known that the hippocampus is part of a larger network and receives input from different brain regions like the cortex. Neurodegeneration due to AD does not only occur in the hippocampus, it is also found in the basal forebrain for example. Therefore, future research is needed which includes the hippocampal-cortical interactions during memory-demanding tasks. This brings us to another limitation of this study, the OFT is in general more used to study novelty-seeking behavior and is not a memory-demanding maze. Therefore, more research is needed in a more memory-demanding maze during the pre-plaque stage to obtain stronger results for learning impairment in the TgF344-AD rats. Electrophysiological acquisitions during more demanding memory tasks at pre-plaque stages in TgF344-AD rats could be valuable to link the observed alterations in PAC to cognitive impairments.

Electrophysiological measurements are prone to artifacts due to non-biological sources such as electrical interference, which could hamper the detection of SWR and the interpretation of the results (Liu et al., 2022). The current study was performed in freely moving animals in the absence of a Faraday cage. However, the W2100 recording headstages are directly connected to the electrode and a small battery was placed on top of the wireless headstage as a power source, which minimizes noise and therefore, abolishes the need for extra shielding. However, also biological sources of noise such as excessive movement and scarring of the brain tissue around the electrode could interfere with the SWR analysis. Two TgF344-AD rats were excluded from the SWR analysis, since we could not detect robust ripples in these animals.

Due to limitations in the experimental setup, the sample size of this study is relatively small (5 animals per group), and it only included male animals. Therefore, future studies should be done with larger sample sizes and a combination of male and female animals.

## 5. Conclusion

In the current study we aimed to investigate how hippocampal oscillations were altered during the pre-plaque stage of AD in TgF344-AD rats. We observed state dependent alterations in theta power and decreased theta-gamma modulation. Moreover, SWR characteristics were altered in TgF344-AD rats, with an increased power of the SWR and slow gamma oscillations, together with a decreased duration of SWR. These alterations in hippocampal activity coincided with increased anxiety-like behavior in TgF344-AD rats.

Future research would be valuable to correlate these alterations in hippocampal activity to cognitive deficits by performing electrophysiological experiments during more complex hippocampal-dependent behavioral tasks. Moreover, pharmacological manipulations could unravel pathological mechanisms behind the observed oscillatory alterations, which could be valuable targets for therapeutic treatment.

## Data availability statement

The original contributions presented in the study are included in the article/Supplementary material, further inquiries can be directed to the corresponding authors.

## Ethics statement

All procedures were in accordance with the guidelines approved by the European Ethics Committee (decree 2010/63/EU) and were approved by the Committee on Animal Care and Use at the University of Antwerp, Belgium (approval number: 2019-06).

## Author contributions

MB, MV, and GK designed the study. GK and MB optimized the surgeries to implant the laminar electrodes and the recordings while the animals were exploring the open field. MB performed the surgeries and acquisition of the electrophysiology and behavioral data. MB, DT, GK, and MV optimized and performed the data analysis and interpretation and wrote the manuscript. MB and DT performed the histological analyses to validate the electrode position. GK and MV supervised the study and supported the study with equipment and materials. All authors provided comments and intellectual input that led to the final version of the manuscript, read and approved the final manuscript.

## Funding

This study was supported by the Fund of Scientific Research Flanders (FWO-G048917N and FWO-G045420N) and the Stichting Alzheimer Onderzoek (SAO-FRA-20180003). The computational resources and services used in this work were provided by the HPC core facility CalcUA of the University of Antwerp, the VSC (Flemish Supercomputer Center), funded by the Hercules Foundation and the Flemish Government department EWI.

## Conflict of interest

The authors declare that the research was conducted in the absence of any commercial or financial relationships that could be construed as a potential conflict of interest.

## Publisher’s note

All claims expressed in this article are solely those of the authors and do not necessarily represent those of their affiliated organizations, or those of the publisher, the editors and the reviewers. Any product that may be evaluated in this article, or claim that may be made by its manufacturer, is not guaranteed or endorsed by the publisher.

## Footnotes

^1^ www.graphpad.com

## Abbreviations

AD: Alzheimer’s disease
Aβ: amyloid-beta
EEG: electroencephalography
Hz: Hertz
LMM: linear mixed model
LFP: local field potential
MCI: mild cognitive impairment
MI: modulation index
OFT: open field test
PAC: phase amplitude coupling
SWR: sharp wave-ripple
WI: wake immobility
PSD: peak spectral density
