# Supplementary material for: Alterations in theta-gamma coupling and sharp wave-ripple, signs of prodromal hippocampal network impairment in the TgF344-AD rat model
Source: Front Aging Neurosci. 2023 Mar 22;15:1081058. doi: 10.3389/fnagi.2023.1081058 (PMC10075364; doi:10.3389/fnagi.2023.1081058)
Supplement: Supplementary file 1 [file Data_Sheet_1.pdf]

Supplementary Material

1.1 Supplementary Figures

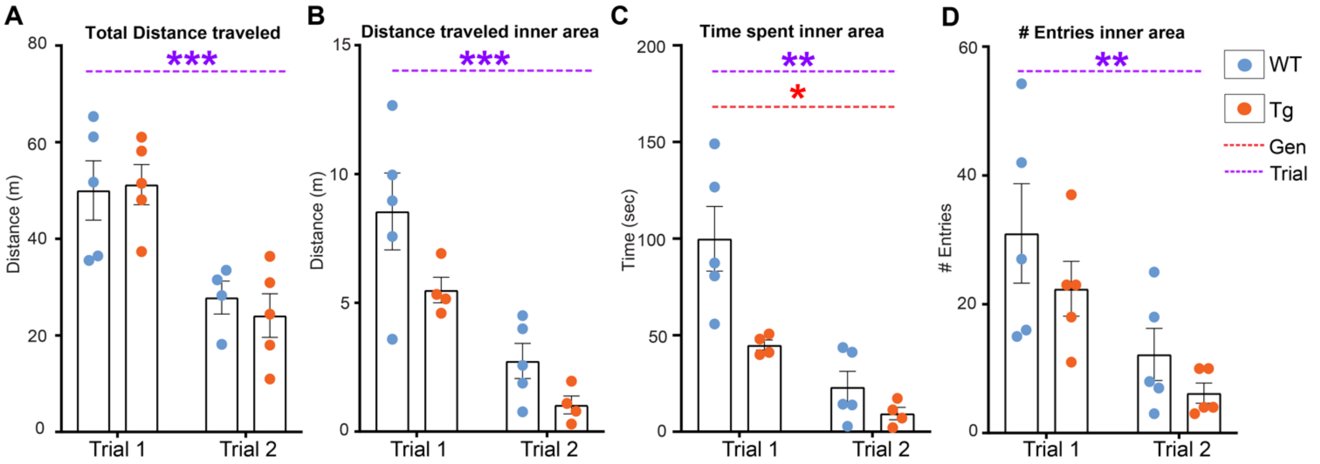

Supplementary figure 1: Behavioral analysis OFT across trials. A) Total distance traveled in meters across trials. B) Distance traveled in the inner area in meters across trials. C) Time spent in seconds in the inner area of the open field across trials. D) Number of entries of the inner area across trials. Bars represent mean +/- SEM, individual values are represented by dots. *p < 0.05 **p = < 0.01, ***p = < 0.001.

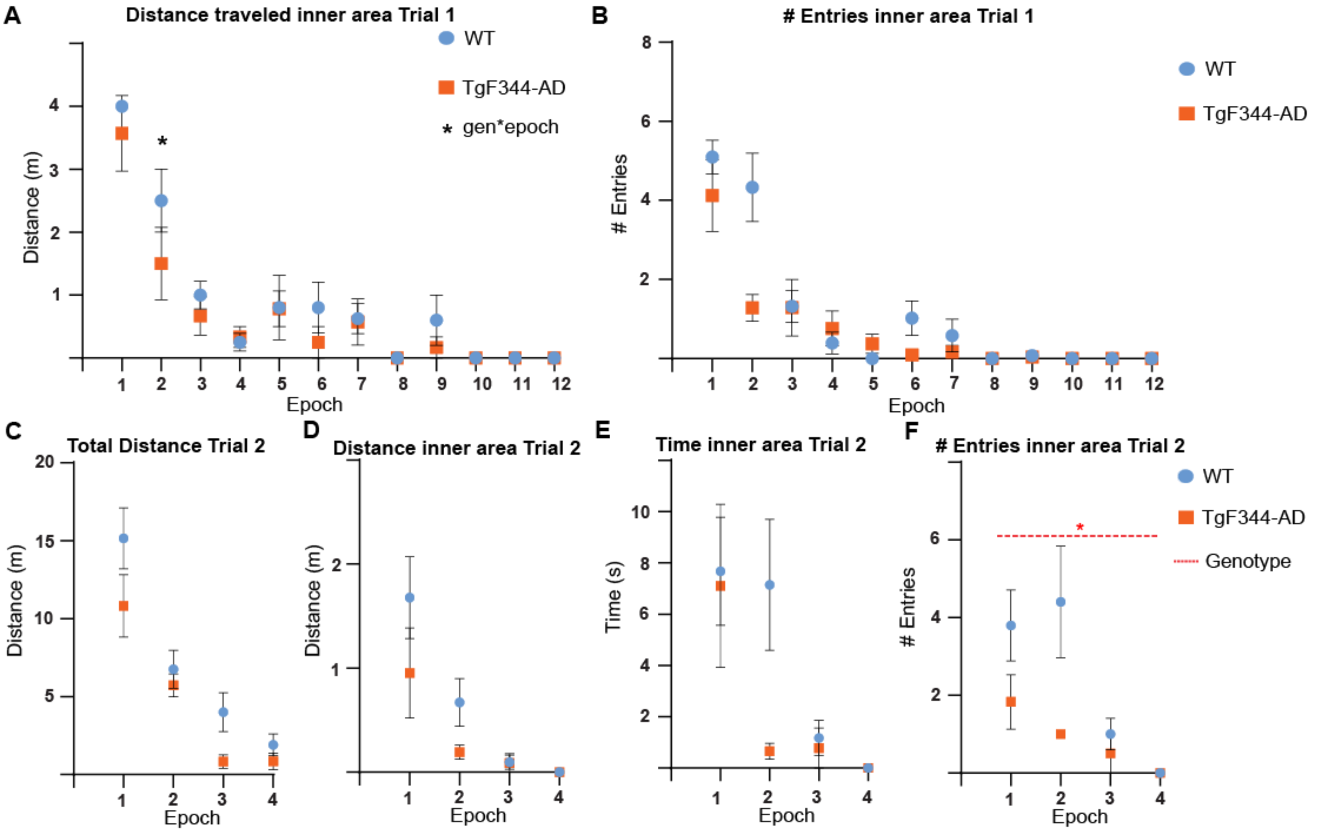

Supplementary figure 2: Behavioral analysis OFT across 5-minute epochs. A) Total distance traveled in the inner area during trial one across different epochs. B) Number of entries into the inner area during trial one across the different epochs. Total distance traveled (C), distance traveled in inner area (D), time spent in the inner area (E) and number of entries in the inner area (F) during trial 2 across epochs. Figures show mean +/- SEM. *p = < 0.05

Supplementary Tables

Supplementary table 1: Results of the linear mixed model (LMM) for the power spectral density of different frequency bands

	Delta			*Theta low*			*Theta high*			*Slow gamma*			*Fast gamma*			*HFO*		
	DF	*F-value*	*p*	*DF*	*F-value*	*p*	*DF*	*F-value*	*p*	*DF*	*F-value*	*p*	*DF*	*F-value*	*p*	DF	F-value	p
Genotype	1,8	2.2555	0.1714	1,8	0.1898	0.6745	1,7.3	5.6648	0.0474	1,8	2.2608	0.1711	1,8	0.7627	0.4078	1,7.9	0.0651	0.8051
Trial	1,20.3	0.4927	0.4907	1,22.3	0.0022	0.9632	1,22.7	0.2756	0.6047	1,24	1.1315	0.298	1,22.2	1.1971	0.2856	1,21.9	1.2224	0.2809
State	1,21.0	5.5623	0.0281	1,22.5	10.649	0.0035	1,22.7	5.5354	0.0277	1,24	15.9465	0.0005	1,22	7.4662	0.0121	1,22.2	1.8178	0.1912
*Tr*Gt*	1,20.3	0.0002	0.9884	1,22.3	1.0658	0.313	1,22.7	1.4248	0.2449	1,24	0.2427	0.6267	1,22.2	0.3244	0.5747	1,21.9	6.3981	0.0191
*St*Gt*	1,21.0	2.144	0.158	1,22.5	0.3058	0.5857	1,22.7	1.884	0.1833	1,24	8.0755	0.009	1,22	9.9745	0.0046	1,22.2	0.4426	0.5128
*St*Tr*	1,20.3	0.0004	0.9852	1,22.5	1.1437	0.2962	1,22.7	1.4127	0.2469	1,24	2.0051	0.1696	1,22	4.8906	0.0377	1,21.9	1.5246	0.23
*St*Tr*Gt*	1,20.3	0.038	0.8474	1,22.5	0.0262	0.8729	1,22.7	0.5847	0.4523	1,24	0.6061	0.4439	1,22	0.0334	0.8566	1,21.9	0.8637	0.3628

DF = degrees of freedom

Supplementary table 2: post-hoc analysis of the power of gamma oscillations

	Slow gamma				*Fast gamma*			
	q-values	*Difference*	*Lower 95%*	*Upper 95%*	*q-values*	*Difference*	*Lower 95%*	*Upper 95%*
WT E – WT QW	0.4235	-0.00088	-0.0031	0.001346	0.8272	0.000045	-0.00026	0.000348
WT E – TG E	0.4235	-0.00356	-0.0124	0.005279	0.8272	-0.00012	-0.00133	0.001092
WT QW – TG QW	0.148	-0.00789	-0.01673	0.000949	0.3449	-0.00079	-0.002	0.000419
TG E – TG QW	<0.0001	-0.00521	-0.00743	-0.00298	0.0021	-0.00063	-0.00095	-0.00031

FDR corrected p-values (q-value), difference of means and boundaries of the confidence interval of the post hoc tests.

Supplementary table 3: post-hoc analysis of the power of HFO

	q-values	*Difference*	*Lower 95%*	*Upper 95%*
WT trial 1 – WT trial 2	0.0587	-0.00013	-0.00022	-2.7E-05
WT trial 1 – TG trial 1	0.5751	-0.00013	-0.00047	0.000219
WT trial 2 – TG trial 2	0.7506	0.00005	-0.00029	0.000393
TG trial 1 – TG trial 2	0.5751	0.000049	-5.5E-05	0.000153

FDR corrected p-values (q-value), difference of means and boundaries of the confidence interval of the post hoc tests.

Supplementary table 4: Results of LMM of the PAC for each behavioral state

	Exploration 35-45 Hz			*Exploration 55-90 Hz*			*Wake immobility 60-90 Hz*		
	DF	*F-value*	*p*	*DF*	*F-value*	*p*	*DF*	*F-value*	*p*
Genotype	1,8.1	0,4303	0,53	1,8.0	0,9149	0,3659	1,5.6	8,5846	0,0284
Trial	1,7.2	42,4208	0,0003	1,8.2	0,0269	0,8737	1,7.2	0,5439	0,484
*Tr*Gt*	1,7.2	9,2711	0,0181	1,7.4	1,5251	0,2547	1,6.1	5,4318	0,0579

DF = degrees of freedom

Supplementary table 5: post-hoc analysis of the PAC during exploration (35-45 Hz).

	q-values	*Difference*	*Lower 95%*	*Upper 95%*
WT trial 1 – WT trial 2	0.0009	-0.003981	-0.00537	-0.0026
WT trial 1 – TG trial 1	0.05743	-0.00166	-0.00323	-8.9E-05
WT trial 2 – TG trial 2	0.2497	0.000877	-0.00069	0.002448
TG trial 1 – TG trial 2	0.05743	-0.001445	-0.00283	-5.9E-05

FDR corrected p-values (q-value), difference of means and boundaries of the confidence interval of the post hoc tests.

Supplementary table 6: Results of the LMM for the Sharp wave ripples per trial.

	PSF SWR			*PSF SG*			*Power SWR*			*Power SG*			*Duration*		
	DF	*F-value*	*p*	*DF*	*F-value*	*p*	*DF*	*F-value*	*p*	*DF*	*F-value*	*p*	*DF*	*F-value*	*p*
Genotype	1,8.2	1.5917	0.2419	1,7.9	1.8032	0.2168	1,7.7	23.9689	0.0013	1,7.8	8.9812	0.0177	1,8.6	0.7627	0.1497
Trial	1,22.5	0.1071	0.7465	1,21.4	5.0894	0.0346	1,7.4	0.0001	0.9914	1,6.3	14.3034	0.0082	1,23.5	1.1971	0.8577
State	1,22.6	122,404	0.002	1,21.4	0.7252	0.4039	1,15	3.3151	0.0887	1,14.1	6.5201	0.0229	1,23.5	7.4662	0.4577
*Tr*Gt*	1,22.7	0.6375	0.433	1,21.4	2.8935	0.1034	1,7.4	0.976	0.3546	1,6.3	0.5822	0.4729	1,23.5	0.3244	0.1275
*St*Gt*	1,22.8	0.6335	0.4344	1,21.4	1.2239	0.2809	1,15	0.1357	0.7177	1,14.1	6.6565	0.0217	1,23.5	9.9745	0.1602
*St*Tr*	1,22.9	2.2521	0.1474	1,21.3	0.0557	0.8158	1,15	0.2619	0.6162	1,14.1	1.1115	0.3095	1,23.5	4.8906	0.8599
*St*Tr*Gt*	1,22.10	3.4823	0.0751	1,21.3	0.0305	0.8631	1,15	0.0774	0.7847	1,14.1	4.7884	0.046	1,23.5	0.0334	0.0286

DF = degrees of freedom

Supplementary table 7: Results of the LMM for the Sharp wave ripples occurrence rate per trial.

	SWR ratio E/WI		
	DF	*F-value*	*p*
Genotype	1,7.0	0.1823	0.6822
Trial	1,7.0	6.8833	0.0342
*Tr*Gt*	1,7.0	2.3741	0.1673

DF = degrees of freedom

Supplementary table 8: post-hoc analysis duration SWR

	q-values	*Difference*	*Lower 95%*	*Upper 95%*
WT-E trial 1 – WT-WI trial 1	0.8758	0.7497	-9.0676	10.5669
TG-E trial 1 – TG-WI trial 1	0.9730	-3.5788	-14.0265	6.869
WT-E trial 2 – WT-WI trial 2	0.1884	-11.5697	-22.0174	-1.1219
TG-E trial 2 – TG-WI trial 2	0.3703	6.99	-2.8272	16.8072
WT-E trial 1 – TG-E trial 1	0.9957	2.0681	-7.553	11.6893
WT-WI trial 1 – TG-WI trial 1	1	-2.2603	-12.5375	8.0169
WT-E trial 2 – TG-E trial 2	0.8018	-1.625	-11.2461	7.9962
WT-WI trial 2 – TG-WI trial 2	0.0252	16.9347	6.6575	27.2119
WT-E trial 1 – WT-E trial 2	0.8421	1.8396	-7.9776	11.6568
TG-E trial 1 – TG-E trial 2	0.9328	-1.8535	-11.6707	7.9637
WT-WI trial 1 – WT-WI trial 2	0.1976	-10.4797	-20.9275	-0.032
TG-WI trial 1 – TG-WI trial 2	0.2940	8.7153	-1.7325	19.163

FDR corrected p-values (q-value), difference of means and boundaries of the confidence interval of the post hoc tests.

Supplementary table 9: post-hoc analysis slow gamma power during SWR

	q-values	*Difference*	*Lower 95%*	*Upper 95%*
WT-E trial 1 – WT-WI trial 1	0.1617	-0.01583	-0.03564	0.003981
TG-E trial 1 – TG-WI trial 1	0.1024	-0.01969	-0.0395	0.000124
WT-E trial 2 – WT-WI trial 2	0.1772	0.016093	-0.00545	0.037636
TG-E trial 2 – TG-WI trial 2	0.0496	-0.03085	-0.05397	-0.00774
WT-E trial 1 – TG-E trial 1	0.1107	-0.01674	-0.03458	0.001094
WT-WI trial 1 – TG-WI trial 1	0.0762	-0.0206	-0.03844	-0.00276
WT-E trial 2 – TG-E trial 2	0.4047	0.00884	-0.01112	0.028796
WT-WI trial 2 – TG-WI trial 2	0.0096	-0.03811	-0.0588	-0.01741
WT-E trial 1 – WT-E trial 2	0.3346	-0.00797	-0.02298	0.007043
TG-E trial 1 – TG-E trial 2	0.1181	0.017616	0.000065	0.035167
WT-WI trial 1 – WT-WI trial 2	0.0488	0.023955	0.006445	0.041466
TG-WI trial 1 – TG-WI trial 2	0.4093	0.006452	-0.0096	0.022503

FDR corrected p-values (q-value), difference of means and boundaries of the confidence interval of the post hoc tests.

Supplementary table 10: Results of LMM for the behavior during the open field test per trial.

	Total distance travelled			*Distance travelled inner area*			*Time spent inner area*			*# Entries inner area*		
	DF	*F-value*	*p*	*DF*	*F-value*	*p*	*DF*	*F-value*	*p*	*DF*	*F-value*	*p*
Genotype	1,8.1	0.0647	0.8055	1,8.5	4.9829	0.0542	1,8.4	9.047	0.0187	1,8.0	1.7344	0.2243
Trial	1,9.2	24.295	0.0008	1,8.8	37.0639	0.0002	1,9.1	30.868	0.0007	1,9.0	19.3489	0.0017
*Tr*Gt*	1,8.3	0.2119	0.6572	1,7.8	0.7627	0.4086	1,8.3	4.2217	0.0729	1,8.0	0.0961	0.7645

DF = degrees of freedom

Supplementary table 11: Results of LMM for the behavior during the OFT across epochs during trial 1

	Total distance travelled			*Distance travelled inner area*			*Time spent inner area*			*# Entries inner area*		
	DF	*F-value*	*p*	*DF*	*F-value*	*p*	*DF*	*F-value*	*p*	*DF*	*F-value*	*p*
Genotype	1,1	0.1308	0.7251	1,10.2	2.2608	0.1629	1,9.6	37.0991	0.0001	1,9.8	1.0292	0.3348
Epoch	11,111.50	40.7545	0.0001	11,96.4	27.2407	0.0001	11,97.2	20.3168	0.0001	11,108.50	28.9335	0.0001
*Ep*Gt*	11,100.50	1.8695	0.0523	11,96.4	2.8359	0.003	11,97.2	6.1843	0.0001	11,97.5	0.5487	0.8651

DF = degrees of freedom

Supplementary table 12: post-hoc analysis for the distance travelled in the inner area during the OFT across epochs during trial 1

Genotype-Epoch	*q-values*	*Difference*	*Lower 95%*	*Upper 95%*

WT1 - TG1	0.5328	0.4854	-0.075	1.0458
WT2 - TG2	0.0012	1.5116	0.93259	2.09061
WT3 - TG3	0.9705	0.01117	-0.58698	0.60931
WT4- TG4	1	-0.1909	-0.78905	0.40724
WT5 - TG5	1	-0.18973	-0.80565	0.42619
WT6 - TG6	0.5252	0.4438	-0.13495	1.02254
WT7 - TG7	1	0.20385	-0.41207	0.81978
WT8 - TG8	1	-0.01423	-0.62991	0.60144
WT9 - TG9	1	0.01377	-0.60215	0.62969
WT10 - TG10	1	-0.0114	-0.59041	0.56761
WT11- TG11	1	-0.0114	-0.59041	0.56761
WT12 - TG12	1	-0.02307	-0.60182	0.55568

FDR corrected p-values (q-value), difference of means and boundaries of the confidence interval of the post hoc tests.

Supplementary table 13: post-hoc analysis for the time spent in the inner area during the OFT across epochs during trial 1

Genotype-Epoch	*q-values*	*Difference*	*Lower 95%*	*Upper 95%*
WT1 - TG1	0.0012	10.3657	5.2502	15.4812
WT2 - TG2	0.0006	21.237	15.5842	26.8899
WT3 - TG3	0.0087	8.1463	2.8513	13.4413
WT4- TG4	0.3485	3.6553	-2.0057	9.3163
WT5 - TG5	0.2932	-4.0457	-9.5315	1.4401
WT6 - TG6	0.0729	5.863	0.568	11.1579
WT7 - TG7	0.0024	9.4627	4.1685	14.757
WT8 - TG8	1	-0.0394	-5.6929	5.6141
WT9 - TG9	1	-0.1963	-5.8491	5.4565
WT10 - TG10	1	0.0161	-5.2782	5.3103
WT11- TG11	1	0.0161	-5.2782	5.3103
WT12 - TG12	0.9981	0.0063	-5.2887	5.3013

FDR corrected p-values (q-value), difference of means and boundaries of the confidence interval of the post hoc tests.

Supplementary table 14: Results of LMM for the behavior during the OFT across epochs during trial 2

	Total distance travelled			*Distance travelled inner area*			*Time spent inner area*			*# Entries inner area*		
	DF	*F-value*	*p*	*DF*	*F-value*	*p*	*DF*	*F-value*	*p*	*DF*	*F-value*	*p*
Genotype	1,10.1	4.61	0.057	1,9.7	2.2094	0.169	1,9.2	1.445	0.2593	1,9.3	6.9639	0.0263
Epoch	3,32.5	41.9369	0.0001	3,27.7	13.1929	0.0001	3,25.9	7.4003	0.001	3,28.9	7.5719	0.0007
*Ep*Gt*	3,29.4	1.0286	0.3942	3,27.8	1.1961	0.3295	3,25.9	1.7281	0.1859	3,25.3	2.9193	0.0535

DF = degrees of freedom
